# Total neoadjuvant therapy for locally advanced rectal cancer: barriers to implementation in real-world practice

**DOI:** 10.1007/s00432-025-06412-6

**Published:** 2026-01-08

**Authors:** David Rene Steike, Niklas Benedikt Pepper, Stefan Gravemeyer, Anne Bremer, Bernhard Glasbrenner, Matthias Brüwer, Andreas Pascher, Dirk Domagk, Lothar Biermann, Philipp Lenz, Hans Theodor Eich, Gabriele Reinartz

**Affiliations:** 1 Department of Radiotherapy – Radiooncology, University Hospital Muenster, Münster, Germany; 2 West German Cancer Center, University Hospital Muenster, Münster, Germany; 3 Department of Hematology – Oncology, St. Franziskus Hospital Muenster, Münster, Germany; 4 Department of Gastroenterology, Diabetology, St. Franziskus Hospital Muenster, Münster, Germany; 5 Department of General and Visceral Surgery, St. Franziskus Hospital Muenster, Münster, Germany; 6 Department of General, Visceral and Transplantation Surgery, University Hospital Muenster, Münster, Germany; 7 Department of Medicine I, Josephs-Hospital Warendorf, Warendorf, Germany; 8 Department of Visceral and General Surgery, Josephs-Hospital Warendorf, Warendorf, Germany; 9 Department of Palliative Care, Institute of Palliative Care, University Hospital of Muenster, Münster, Germany

**Keywords:** Locally advanced rectal cancer, Total neoadjuvant therapy, TNT, Real-world evidence, Organ preservation, Multimodal therapy

## Abstract

**Purpose:**

Total neoadjuvant therapy (TNT) has demonstrated superior oncologic outcomes and improved organ preservation in randomized trials for locally advanced rectal cancer (LARC). This study aimed to assess the real-world implementation of TNT in clinical routine and identify barriers to its application.

**Methods:**

This retrospective, multicenter cohort study analyzed 111 patients with LARC treated between February 2021 and May 2024 across three certified colorectal cancer centers in Germany. Patients were stratified into TNT and non-TNT groups according to their neoadjuvant treatment modality. Primary endpoints included treatment adherence, disease-free survival (DFS), postoperative morbidity, and documented reasons for omitting TNT. Outcomes were compared with benchmark protocols from established TNT trials.

**Results:**

Despite fulfilling guideline criteria, only 23 of 111 patients (20%) received TNT. Main barriers to TNT included advanced age, comorbidities, and patient refusal. The 1-year DFS rate was 96% in the TNT group and 94% in the non-TNT group. Severe low anterior resection syndrome (LARS) occurred in 9% of TNT patients compared to 22% in the non-TNT group. Postoperative complication rates were similar across both groups. While TNT was associated with favorable trends in disease control and functional outcomes, statistical significance was not reached.

**Conclusion:**

There is a notable discrepancy between clinical guideline recommendations and real-world TNT implementation in LARC. Addressing patient-specific barriers and adopting standardized, risk-adapted treatment decision frameworks may improve clinical integration of TNT, particularly in older and comorbid populations.

## Introduction

The management of locally advanced rectal cancer involves a multimodal approach, integrating systemic therapy, radiotherapy, and surgical intervention. Since 1985, combined chemoradiotherapy (CRT) has played a significant role in reducing pelvic recurrence and distant metastasis, while also enhancing overall survival (OS) (Gastrointestinal Tumor Study Group 1985; Wee et al. 2018; Vendrely et al. 2022).

In recent years, total neoadjuvant therapy (TNT)—administering both chemotherapy and radiotherapy before surgery—has emerged as a preferred strategy, especially for high-risk patients. Trials including RAPIDO (Bahadoer et al. 2021), CAO/ARO/AIO-12 (Fokas et al. 2022), PRODIGE-23 (Conroy et al. 2021), and OPRA (Verheij et al. 2024)have shown that TNT improves pathological complete response (pCR) rates and reduces distant metastases. While most studies including TNT did not demonstrate significant differences in OS, more recent data, such as the 7-year results of the PRODIGE-23 trial (Conroy et al. 2024)and the 3-year results of the STELLAR trial (Jin et al. 2022), now show a significant improvement in overall survival with TNT. Moreover, the treatment concepts varied in timing, sequencing, and intensity of components, ranging from induction chemotherapy (PRODIGE-23) to consolidation chemotherapy (OPRA), and from short-course radiotherapy (RAPIDO, Polish II (Bujko et al. 2016), Trans-Tasman (Ngan et al. 2012)) to long-course CRT (CAO/ARO/AIO-12).

As a result, the current therapeutic landscape is characterized by clinical heterogeneity, despite consistent guideline recommendations. The latest NCCN Guidelines (v1.2025) (National 2025) designate TNT as a Category 1 treatment option for stage II–III locally advanced rectal cancer (LARC) with high-risk features, such as T4 tumors, N2 status, extramural venous invasion (EMVI+), or threatened mesorectal fascia (MRF+). In the ESMO Clinical Practice Guidelines by Hofheinz et al., total neoadjuvant therapy (TNT) is explicitly recommended as the preferred treatment option for locally advanced, MRI-defined rectal cancer (Hofheinz et al. 2025). Despite these recommendations, real-world adoption of TNT remains inconsistent across institutions and patient populations. Factors such as comorbidities, frailty, logistical limitations, and patient preference often influence decision-making—elements that are underrepresented in clinical trials.

In this retrospective, multicenter study, we analyzed 111 patients treated for LARC at three certified colorectal cancer centers in western Germany. The aim of this study is to assess the use and outcomes of different multimodal treatment strategies in clinical routine and to identify reasons for deviation from evidence-based protocols.

## Methods

### Study design and therapy concepts

111 patients with tumors located in the middle and lower two thirds of the rectum, classified as UICC stage II or III (T3/T4 tumors and/or lymph node-positive) from the University Hospital of Muenster, Josephs-Hospital Warendorf and Franziskus Hospital Muenster, treated between February 2021 and May 2024, were retrospectively analyzed.

In all cases, the preferred neoadjuvant treatment regimen was determined by consensus in the respective interdisciplinary tumor board by representatives of the certified colorectal cancer centers. Following completion of neoadjuvant (chemo-)radiotherapy and TNT, re-staging was performed after 2–4 weeks, followed by TME or W&W, again based on tumor board consensus. All patients provided written informed consent permitting the use of pseudonymized clinical data for retrospective research purposes. Data sharing between the participating institutions was regulated by an agreement authorized by the local data protection board. The analysis was conducted in accordance with institutional regulations and the principles of the Declaration of Helsinki.

Patients were treated with a neoadjuvant chemoradiotherapy up to a total dose of 50.4 Gy with single fractions of 1.8 Gy and concomitant chemotherapy with 5-fluorouracil (5-FU) or capecitabine. Alternatively, short-term radiotherapy without simultaneous chemotherapy with 5 × 5 Gy was performed.

RT data was gathered from the database of the institution’s Department of Radiation Oncology at Muenster University Hospital, Germany. The irradiation technique for all 111 patients was intensity modulated radiotherapy (IMRT) using Halcyon^Tm^ or TrueBeam^Tm^ linear accelerators (Varian Medical Systems, Palo Alto, CA, USA) for five daily fractions per week. In the target volume definition, the iliac lymph nodes (interna, externa and communis) and the presacral lymph nodes were included. For N+, the level of L4/5 was defined as the upper field limit. In the case of N0, the cranial boundary was the promontorium.

## Consensus-based treatment strategy for locally advanced rectal cancer

Due to the improved complete pathological response in the 5 × 5 Gy arm of the RAPIDO study (28.4% vs. 14.3% in the standard chemoradiotherapy arm) and the improved disease free survival (DFS) compared to standard therapy (30.4% vs. 23.7%, hazard ratio 0.75, *p*= 0.019) (Bahadoer et al. 2021), it was agreed to proceed analogously to the RAPIDO protocol for TNT implementation. After publication of the 5-year data of the RAPIDO trial and the result of a significant deterioration in the local control rate (10.2% vs. 6.1%, *p*= 0.03) (Dijkstra et al. 2023), a reassessment of recommended concepts was carried out, so that from 2024 total neoadjuvant CRT with 5-FU and subsequent chemotherapy in orientation to the ongoing ACO/ARO/AIO-18.1 trial was preferred.

### Grading side effects, postoperative complication and follow-up

Radiotherapy-related side effects were graded using CTCAE v5.0; postoperative complications were assessed per a modified Clavien-Dindo classification. Complications were categorized by severity from mild (Grade 1) to life-threatening (Grade 4). All patients underwent quarterly clinical follow-up after treatment.

### Statistical analyses and outcome measures

General descriptive statistics were calculated using IBM SPSS Statistics 24 (IBM Corporation, Somers, NY, USA) and GraphPad Prism 9.1 (GraphPad Software, San Diego, CA, USA). Graphical representations were created with GraphPad Prism 9.1, OriginPro 2021b (OriginLab Corporation, Northampton, MA, USA). For nominal variables (TNT vs. non-TNT), the analysis relied on the Chi-square test and Fisher’s Exact test with the latter applied in cases of particularly small sample sizes. Tumor stage distribution across the different therapy groups as well as the comparison of post-operative side effects between the different therapy groups were analyzed using the Kruskal–Wallis test. The statistical significance level was set to 0.05 for all analyses.

## Results

### Patient characteristic and treatment outcome

**Fig. 1:**
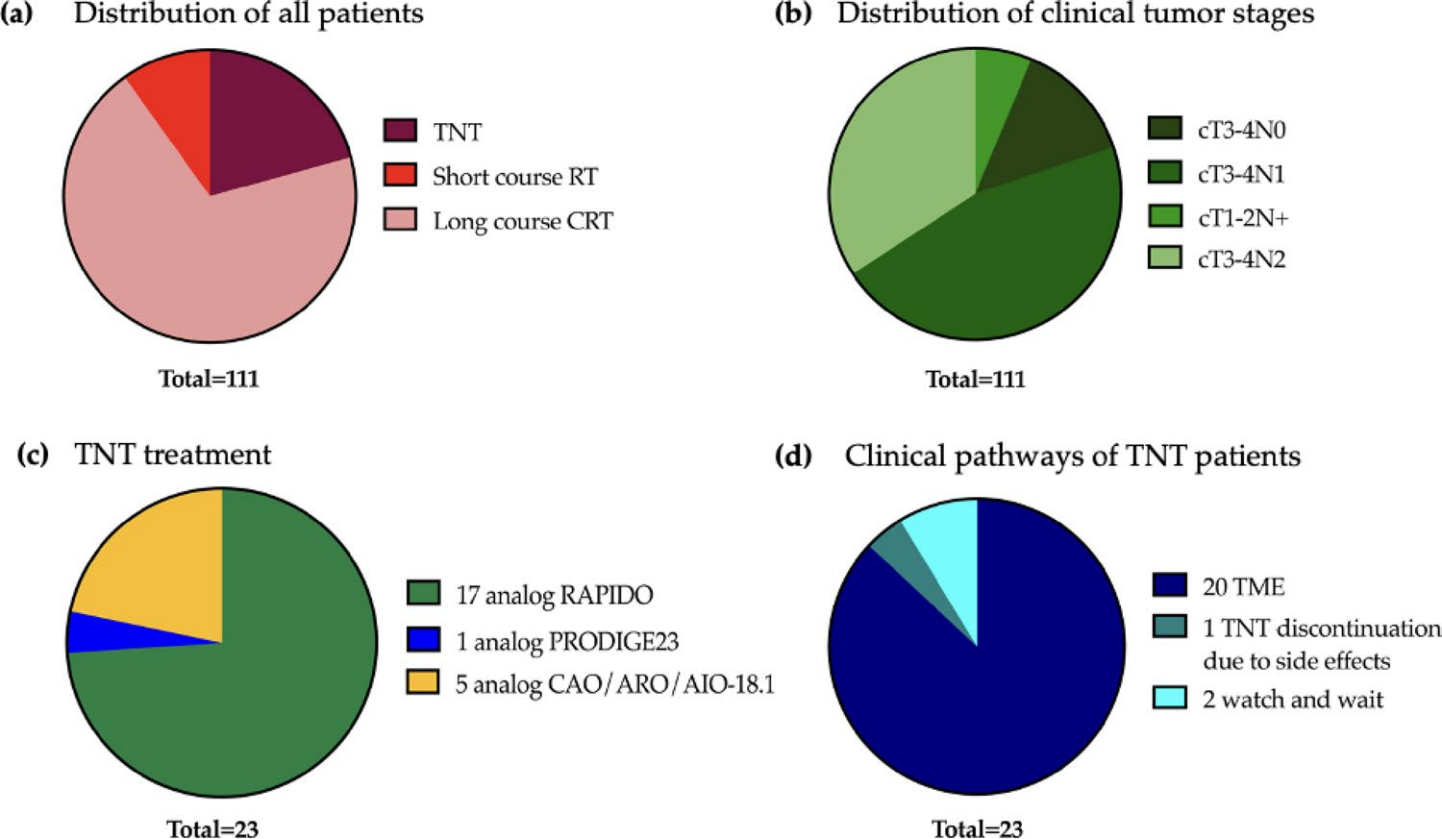
Patients with locally advanced rectal cancer were analyzed. Among the 111 patients, 23 received total neoadjuvant therapy (TNT) (**a**). Most patients had tumor stage cT1-2 N+. The distribution of tumor stages also includes patients with affected mesorectal fascia (MRF+) and/or extramural venous invasion (EMVI+) (**b**). The TNT patients were treated according to three different study-based protocols (**c**), each with distinct treatment sequences. Most TNT patients underwent total mesorectal excision (TME) (**d**). Abbreviations: CRT: Chemoradiotherapy; N+: All patients with node-positive disease; RT: radiotherapy; TME: Total mesorectal excision; TNT: total neoadjuvant therapy; Watch and wait: non-operative management

Although all patients met established high-risk criteria for total neoadjuvant therapy—such as cN2, cT4, cT3 combined with cN1, extramural venous invasion (EMVI+), or mesorectal fascia involvement (MRF+)—only 23 patients (20%) ultimately received TNT (see Fig. 1). In a comparative analysis of patients undergoing TNT versus standard neoadjuvant therapy (non-TNT), several key clinical and treatment-related outcomes were evaluated (s. Table 1). The TNT group (*n* = 23) included patients treated according to RAPIDO, PRODIGE-23, or analogously to the ongoing ACO/ARO/AIO-18.1 trial, based on the chemotherapy regimen applied in that study (6× mFOLFOX or 4× CAPOX for 12 weeks). The non-TNT group (*n* = 88) consisted of those treated only with long-course CRT (50.4 Gy + 5-FU/Capecitabine) or short-course radiotherapy (5 × 5 Gy) before surgical evaluation (see Fig. 1). Statistical analysis did not show a significant impact of TNT vs. non-TNT regarding any of the evaluated end-points (Underwent surgery, R0-resection, Toxicity grade 3 after RT, Toxicity grade 3 after OP, severe LARS, 1-y-DFS, 2-y-DFS), as illustrated in Table 1. However, patients who received TNT demonstrated favorable trends in disease control (1-/2-year disease-free survival, *p* = 1.0/0.67) and functional outcomes.

**Table 1:** Treatment outcomes following TNT versus conventional neoadjuvant therapy (Non-TNT)

Variable	TNT % (95% CI)	Non-TNT % (95% CI)	*P* value
Underwent surgery	96 (78–100)	91 (83–96)	0.68
R0-resection	96 (78–100)	95 (89–99)	1.00
Toxicity grade 3 after RT	9 (1–28)	2 (0–8)	0.11
Toxicity grade 3 after OP	30 (13–53)	32 (22–43)	0.78
Severe LARS	9 (1–28)	23(14–32)	0.23
1-y-DFS	96 (78–100)	94 (87–98)	1.00
2-y-DFS	96 (71–100)	90 (78–96)	0.67

A Chi square Test was performed for nominal variables. None of the differences reached statistical significance, TNT showed favorable trends in disease control and functional outcomes (e.g., lower LARS). (*p* > 0.05). Abbreviations: CI: confidence interval; RT: radiotherapy; OP: operation; LARS: low anterior resection syndrome; 1-/2-y-DFS: disease-free survival after one/two years

Table 2 depicts patient- and toxicity characteristics stratified by applied treatment regime. At baseline, most patients (34%) presented with advanced disease, particularly cT3–4N1–2 stages. The highest proportions of cT3–4N2 cases were observed in the RAPIDO and conventional CRT groups, with 9 and 25 patients, respectively. Patients treated with TNT demonstrated high rates of proceeding to surgery (88–100%) and achieving R0 resection (94–100%), comparable to those in the non-TNT cohort (91% and 96–100%, respectively). Grade 3 postoperative complications were most frequently reported in the conventional CRT group (24 patients), followed by the 5 × 5 Gy group (4 patients) and the RAPIDO arm (6 patients).

**Table 2:** Patient characteristics and treatment outcomes: neoadjuvant therapy with and without TNT

	TNT—RAPIDO	TNT—PRODIGE23	TNT—concept analogous ACO/ARO/AIO-18.1	50.4 Gy + 5-FU/Capec	5 × 5 Gy
Number	17	1	5	77	11
Age (median)	59 (46–71)	51	61 (60–69)	57 (41–86)	58 (51–83)
Sex (M/F)	13/4	1/0	3/2	46/31	5/6
Watch and wait (number)	2	0	0	7	1
Underwent surgery (%)	88	100	100	91	91
R0 (%)	94	100	100	96	100
Toxicity grade 3 after OP (number)	6	0	1	24	4
Recurrent disease (number)	1	0	0	8	1
1-y-DFS (*n* = 111)	94%	100%	100%	92%	100%
2-y DFS (*n* = 74)	94%	100%	–	89%	91%
cT1–2 cN+ (number)	1	0	0	3	3
cT3–4 cN0 (number)	1	1	0	10	3
cT3–4 cN1 (number)	6	0	2	36	4
cT3–4 cN2 (number)	8	0	3	20	1
cT1–3 cN0–2 cM1 (number)	1	0	0	8	0
ypT0 ypN0 cM0 (number)	4	0	2	8	3
ypT1–2 ypN0 cM0 (number)	2	0	1	19	3
ypT3 ypN0 cM0 (number)	6	1		35	3
ypT1–3 ypN1 cM0 (number)	2	0	1 1	3	1
ypT1–3 ypN2 cM0 (number)	0	0	0	1	0
ypT1–3 ypN1–2 cM1 (number)	1	0	0	4	0
pCR after neoadjuvant treatment (number)	4	0	2	8	3
Toxicity grade 3 after RT (number)	2	0	0	2	0
Toxicity grade 3 after OP (number)	6	0	1	24	4

Detailed comparison of patient demographics, tumor staging, treatment outcomes, and toxicity rates across five neoadjuvant therapy regimens in locally advanced rectal cancer. The table contrasts three TNT protocols (RAPIDO, PRODIGE-23, ACO/ARO/AIO-18.1 (6x mFOLFOX or 4x CAPOX) with standard chemoradiotherapy (50.4 Gy + 5-FU/Capecitabine) and short-course radiotherapy (5 × 5 Gy). It includes surgical rates, pathologic outcomes (R0 resection, ypN+), acute toxicity (radiotherapy and postoperative), disease recurrence and DFS rates at one and two years. Tumor stage distribution and the use of non-operative management (watch-and-wait) are also summarized. A watch and wait strategy was used for a total of 10 patients. The DFS differed slightly in the respective groups. All patients who were treated until May 2023 were included in the calculation of the 2-y DFS. Abbreviations: Capec: Capecitabine; RT: radiotherapy; OP: operation; 1-/2-y-DFS: disease-free survival after one/two years.

Severe LARS occurred in 23% of patients in the CRT group (18 of 79 evaluated patients) but was rare among TNT patients, with only two cases observed (9%). LARS was assessed using the validated LARS questionnaire during routine follow-up visits. It was defined as a LARS score of ≥ 30 points (Emmertsen and Laurberg 2012). This typically corresponds to pronounced symptoms such as high stool frequency, severe urgency, incontinence for flatus, unexpected stool leakage, and a substantial impairment of daily activities and quality of life.

Median follow up was 24 months. One-year disease-free survival (DFS) was high across all groups, ranging from 92% to 100%. At two years, DFS remained high in the RAPIDO (94%) and PRODIGE 23 (100%) groups, while slightly lower in the CRT (89%) and 5 × 5 Gy (91%) groups.

In 10 of the 111 cases, a W&W approach was adopted with complete clinical remission (CR). Among them, one patient—who had received long-course chemoradiotherapy without prior TNT—experienced a local recurrence after two years. Of the eight patients presenting with ypN + status following neoadjuvant therapy (3 treated with TNT, 5 treated with CRT), six developed either local recurrence or distant metastasis within two years. Two of these patients were classified as ypN2 status indicating involvement of metastatic cancer in four or more regional lymph nodes.

### Side effects: postoperative complication rates were comparable across treatment groups

**Fig. 2:**
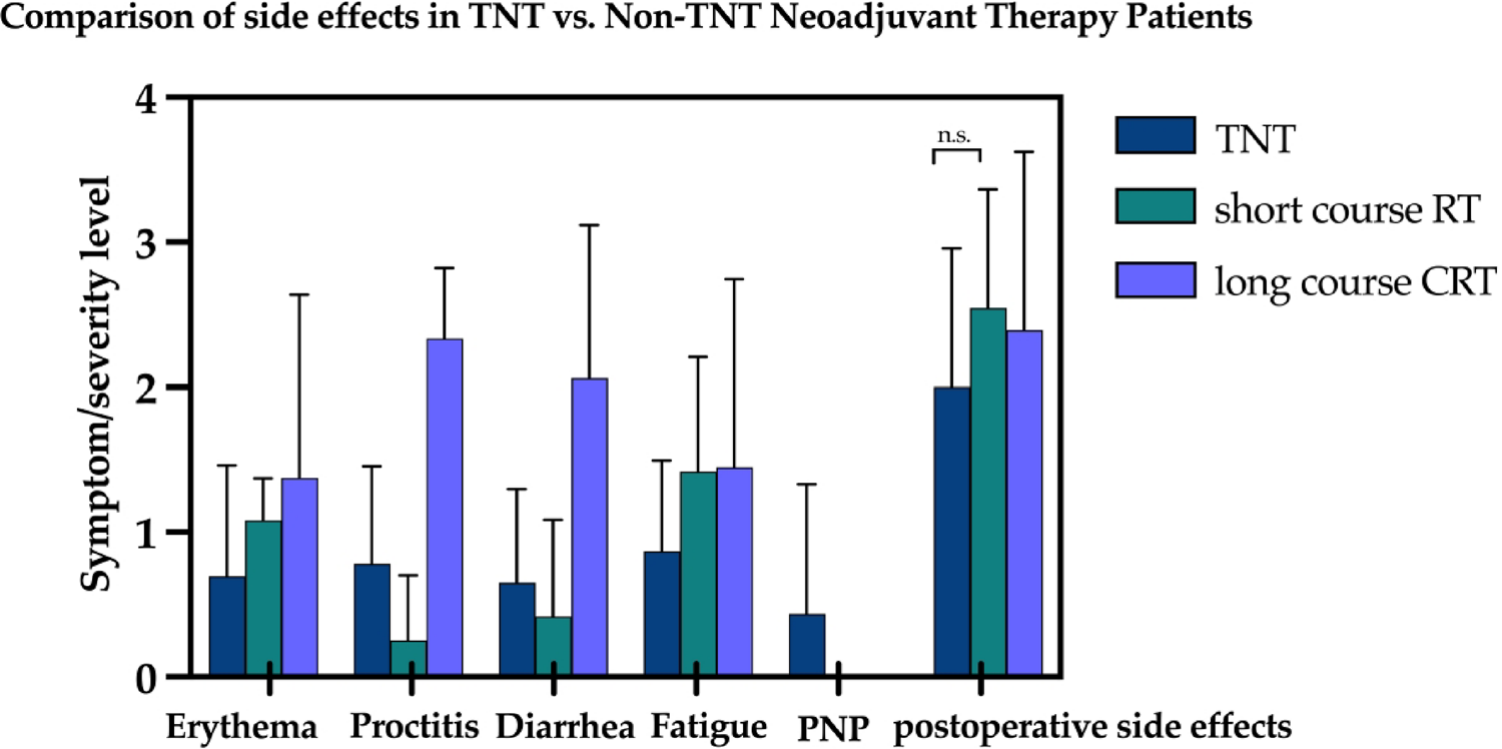
Comparison of side effects between the different treatment concepts. The x-axis lists the respective side effects of the treatment. The y-axis indicates the symptom severity level according to the Common Terminology Criteria for Adverse Events (CTCAE). In particular, there were no significant differences between the TNT group and the neoadjuvant concepts without TNT (non-TNT). Abbreviations: CRT: chemoradiotherapy; PNP: Polyneuropathy; RT: radiotherapy; TNT: total neoadjuvant therapy

The side effect profiles of the three total neoadjuvant therapy regimens (see Fig. 2) were compared with the two conventional neoadjuvant therapy concepts (short-course RT and long-course CRT). Marked differences were observed, particularly in gastrointestinal toxicities. Patients receiving 50.4 Gy + 5-FU/Capecitabine reported the highest severity of proctitis and diarrhea (28% and 36% grade 3), whereas the 5 × 5 Gy group consistently showed the lowest levels of those acute side effects (0% grade 3). Fatigue as well as mild to moderate skin reactions occurred at similar levels across all groups, and peripheral neuropathy was rarely observed. Postoperative complications were pronounced in all three groups, but no statistically significant differences were found between TNT and non-TNT patients. A reduced incidence of a severe LARS with rates of 9% in the TNT and 22% in the non-TNT-group (*p* = 0.23) showed a favorable trend of functional outcome in the TNT group.

#### Reasons why a TNT was not applied

**Fig. 3:**
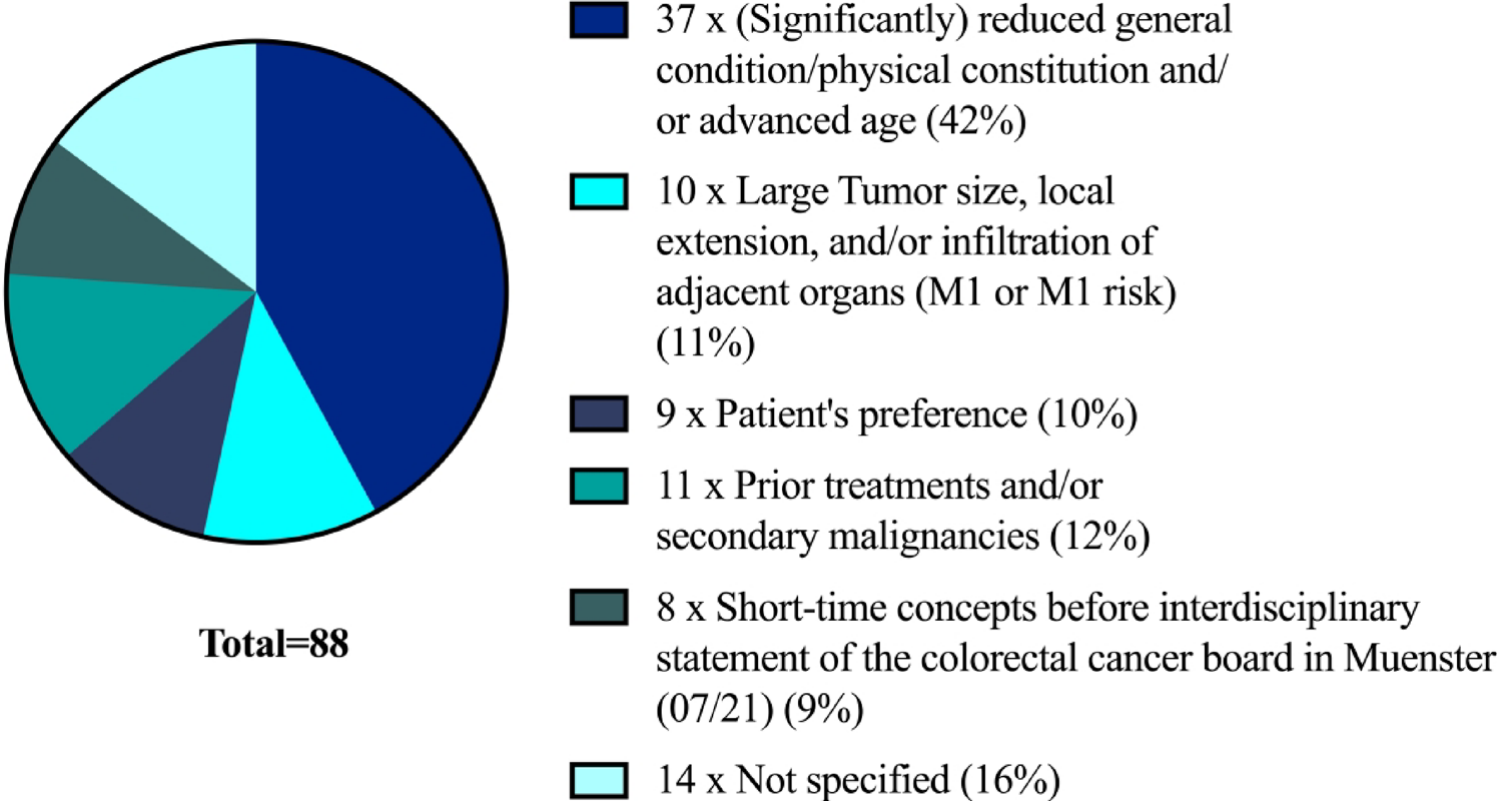
Distribution of reasons against performing a TNT. The most common reason for omitting TNT was a significantly reduced general condition or advanced age (*n* = 88, retrospectively documented reasons from tumorboards, patient documents and doctor’s letters)

In this study, the reasons for not pursuing TNT (*n* = 88) were retrospectively identified based on information from tumor board discussions and written statements provided by the treating physicians (see Fig. 3). While the reasons were clearly documented and explicitly stated in most cases, the rationale was not specified in 14 patients.

In over 42% of cases, TNT was not administered due to a significantly reduced general condition or because of patient age (i.e. 80 years and older). An additional 11% of patients were explicitly excluded based on prior treatments, comorbidities, and a potentially increased risk of complications (e.g. hematological toxicity and diarrhea grade 3 or higher). 10% of patients refused TNT for personal reasons, including fear of side effects or a general rejection of prolonged chemotherapy. In 11 cases, TNT was not performed despite extensive local tumor burden. In these patients, the decision against TNT was primarily based on concerns regarding tolerability and the high risk of early systemic progression. In several of these cases, distant metastases (M1 stage) were detected during subsequent restaging, retrospectively confirming the advanced systemic disease, and explaining the clinical decision to omit TNT in favor of palliative or less intensive treatment approaches.

## Discussion

TNT for locally advanced rectal cancer combines chemotherapy and radiotherapy before surgery and has shown improved pathological response and reduced distant recurrence in multiple trials (Schrag et al. 2023; Sermeus 2014; Ortholan et al. 2012). Its conceptual foundation was laid by the CAO/ARO/AIO-94 trial, which improved local control over postoperative chemoradiotherapy, although distant control remained insufficient (Sauer et al. 2004). In our real-world cohort, only 23 out of 111 patients received TNT as defined by major trials, despite matching eligibility criteria.

This limited uptake reflects a broader implementation gap. Variability in trial protocols and outcomes has led to a fragmented evidence base, making standardization difficult. In our study, we observed that a highly heterogeneous treatment landscape persisted for an extended period even in a confined area with multiple certified colorectal cancer centers, until a formal consensus was reached to adopt a RAPIDO-based TNT protocol.

Smaller real-world studies have also reported favorable TNT outcomes but note significant challenges in implementation (Correia Gomes et al. 2024). In clinical routine, decisions are often shaped by age, comorbidity, and patient preference—factors underrepresented in trials but central to care. Outside major study centers, institutions must balance evidence with feasibility, which can delay or dilute protocol adoption.

The current treatment landscape is characterized by competing strategies—short-course (RAPIDO, Trans-Tasman, Polish II) vs. long-course (CAO/ARO/AIO-12)—without direct comparability (Bahadoer et al. 2021; Bujko et al. 2016; Ngan et al. 2012; Zwart et al. 2022; Fokas et al. 2019). Moreover, the 5-year RAPIDO results showing decreased local control in the TNT group (Dijkstra et al. 2023)have reignited debate on short-course strategies. The forthcoming ACO/ARO/AIO-18.1 results are expected to clarify comparative benefits (Claus 2024).

In parallel, quality of life and organ preservation have become increasingly central in LARC treatment (Sermeus 2014). Trials like OPRA (Verheij et al. 2024), OPERA (Ortholan et al. 2012), TREC and STAR-TREC (Bach et al. 2021; Bach 2023)have established organ preservation as a viable goal—even in advanced disease. A tailored approach, either through escalation via TNT or de-escalation as it is presented in the PROSPECT-study (Schrag et al. 2023), is now at the forefront of modern oncologic care.

Still, the small number of TNT-treated patients in our cohort highlights the need for alternatives. Frail or elderly patients may not tolerate intensified regimens (Van Der Vlies et al. 2021; Schiphorst et al. 2014). While TNT could theoretically provide early systemic control and organ preservation in older individuals (Steinke et al. 2023; Myint and Gérard 2020), practical barriers—frailty, reduced adherence, cognitive decline—limit its feasibility (Steinke et al. 2023; Myint and Gérard 2020; Millan et al. 2015; Papamichael et al. 2015). This is particularly relevant as over 50% of rectal cancer patients are older than 70, yet represent less than 20% of clinical trial participants, according to the Robert Koch Institute (RKI). Our findings align with this mismatch: patients over 80 were excluded from trials such as PRODIGE- 23 (Conroy et al. 2021)or CAO/ARO/AIO-12 (Fokas et al. 2019) and even those eligible often required ECOG 0–1.

Given these constraints, geriatric assessment tools and functional status evaluations (e.g., ECOG) should be standard in tumor boards to enable realistic decision-making. A W&W strategy may be considered in selected patients, particularly those with comorbidities (Haak et al. 2020), although the necessary extent of neoadjuvant treatment before W&W remains unclear.

In our cohort, there was no significant difference in ≥ grade 3 toxicity between TNT and non-TNT groups. However, larger trials report substantial increases in acute toxicity with TNT (e.g., RAPIDO: 48% vs. 25%) (Bahadoer et al. 2021), reinforcing concerns for frail populations. Structured assessments prior to treatment discussions may help better align therapy intensity with individual risk tolerance.

Patient autonomy must also be considered. Nine patients in our study declined TNT due to fear of side effects. This reflects a broader shift in patient values—toward quality of life and organ function. Gani et al. (Gani et al. 2019)showed that many patients are willing to accept increased local recurrence risk in exchange for organ preservation. These preferences highlight the importance of evolving treatment modalities such as brachytherapy (Fleischmann et al. 2024), hyperthermia (Shoji et al. 2015; Wang et al. 2022), and adaptive radiotherapy (Kensen et al. 2023), which may reduce toxicity while maintaining oncologic outcomes.

In our cohort, 37 patients (33%) were deemed unfit for TNT due to age or reduced performance. This supports ongoing trials like OPERA (Gerard et al. 2023)and ACO/ARO/AIO-22 (Fleischmann et al. 2024), which focus on reduced-intensity strategies for vulnerable populations. These innovations expand the treatment toolbox beyond TNT and may become essential as patient demographics continue to shift.

Ourdata underline the need for biological stratification. ypN + status was a predictor of early recurrence, confirming the limited systemic control in certain subgroups. Similar to the role of microsatellite instability (MSI) in immunotherapy (Cercek et al. 2022), further biomarkers may enable tailored treatments beyond anatomical staging. Prospective trials must integrate molecular profiling to refine TNT indications and optimize outcomes.

Heterogeneity across trials also complicates evidence transfer. While our patients met criteria from RAPIDO (cT4, N2, EMVI+, MRF+) and CAO/ARO/AIO-12 (cT3/4, N+), outcomes are difficult to compare due to differing protocols and endpoints. PROSPECT (Schrag et al. 2023), for example, included upper rectal tumors not typically qualifying for neoadjuvant therapy, challenging its external validity. Standardized definitions of LARC and MRI staging criteria are needed to align future studies. Ongoing investigations such as ACO/ARO/AIO-18.2 (Hofheinz 2023), which examines disease-free survival in de-escalated regimens, may contribute critical insights and facilitate more nuanced risk-adapted strategies.

Lastly, structural characteristics of national healthcare systems may influence the implementation of TNT in clinical practice. In Northern European countries, centralized and state-regulated healthcare systems allow complex oncological treatments to be concentrated in a limited number of high-volume centers. Clear national regulations, comprehensive electronic cancer registries, and established quality monitoring frameworks support standardized treatment pathways and consistent outcomes. In Germany, by contrast, the healthcare system is decentralized and characterized by a large number of independent hospitals with strong self-governance. Certifications such as Deutsche Krebsgesellschaft (DKG)-accredited colorectal cancer centers are voluntary. In addition, the fragmented registry landscape limits nationwide outcome monitoring. Alongside the factors discussed above, which represented barriers within our cohort, these structural conditions may contribute to a more heterogeneous implementation of TNT.

### Limitations

This study is limited by its retrospective design and small sample size. The relatively short follow-up period of up to two years may also not fully capture long-term outcomes. Furthermore, the heterogeneity of treatment regimens, even within the TNT cohort complicates direct comparisons. Although efforts were made to align treatments with protocol-based standards, deviations based on institutional practice, physician discretion, or patient preference may have introduced bias. Lastly, functional assessments were not systematically collected using validated patient-reported outcome measures (PROMs), limiting the depth of insight into quality-of-life dimensions. Future multicenter trials with standardized treatment protocols, extended follow-up, and integration of PROMs are warranted to address these limitations and optimize TNT implementation in real-world settings.

## Conclusion

This multicenter retrospective analysis highlights the gap between clinical evidence and real-world implementation of TNT in patients with locally advanced rectal cancer. Despite broad eligibility, only 20% of patients received TNT, primarily due to age, comorbidities, and individual preferences. These findings emphasize that beyond trial data, real-world feasibility—including logistical constraints and patient-centered factors—must be considered in daily oncology practice. To improve TNT integration, structured decision-making frameworks are needed, including routine geriatric assessments and individualized risk-benefit discussions. A clearer understanding of trial criteria, endpoints, and long-term data is essential for evidence-based tumor board decisions, particularly in centers outside major study networks. Future strategies must ensure that vulnerable populations are not excluded from guideline-concordant, yet personalized, care.

## Data Availability

The data that support the findings of this study are available from the corresponding author upon reasonable request.
